# *In vitro* and *in vivo* therapeutic approach for a small cell carcinoma of the ovary hypercalcaemic type using a SCCOHT-1 cellular model

**DOI:** 10.1186/s13023-014-0126-4

**Published:** 2014-08-08

**Authors:** Anna Otte, Finn Rauprich, Peter Hillemanns, Tjoung-Won Park-Simon, Juliane von der Ohe, Ralf Hass

**Affiliations:** Biochemistry and Tumor Biology Laboratory, Department of Gynecology and Obstetrics Medical, University Hannover, Carl-Neuberg-Str. 1, D – 30625 Hannover, Germany

**Keywords:** SCCOHT, Ovarian cancer, Tumor growth, Chemotherapy

## Abstract

**Background:**

The small cell ovarian carcinoma of the hypercalcemic type (SCCOHT) which preferably affects young women during regenerative age represents a rare and aggressive form of ovarian tumors with poor prognosis and lacks an efficient therapy.

**Methods and results:**

*In vitro* chemotherapy testing in a fluorescence assay using a recently developed cellular model from a recurrent SCCOHT revealed sensitivity for certain epothilones, methotrexate and topotecan whereas little if any cytotoxicity was observed with other chemotherapeutics including platin-based compounds. In particular, epothilone B demonstrated a high sensitivity in contrast to ixabepilone with only little detectable effects. Western blot and cell cycle analysis revealed that the epothilone B sensitivity was associated with increased Ser^15^ phosphorylation of p53, a significant G_1_ and G_2_ cell cycle accumulation and subsequent cell death in subG_1_ phase. Moreover, tubulinβ3 expression in SMARCA4/BRG1-defective SCCOHT-1 in contrast to other ovarian cancer cells was also affected during chemotherapy treatment. Increased extracellular Ca^2+^ levels further enhanced the epothilone B cytotoxicity in SCCOHT-1 cells. These *in vitro* effects were also confirmed *in vivo* in NOD/scid mouse xenografts demonstrating an attenuated tumor growth in epothilone B / Ca^2+^-treated mice. After 4d of subsequent treatment, the tumor sizes were reduced by about 90% as compared to continuously growing control tumors. In parallel, a hypercalcemia in control tumor-carrying mice was reverted to normal serum Ca^2+^ levels after epothilone B / Ca^2+^ therapy.

**Conclusions:**

Taken together, these data demonstrated anti-tumorigenic effects of epothilone B / Ca^2+^ in SCCOHT providing a focused therapeutic approach against this rare disease and arising recurrent tumors.

## Background

Ovarian cancer represents one of the most lethal gynecologic malignancy. A rare form of an aggressive ovarian tumor is displayed by the small cell ovarian carcinoma of the hypercalcemic type (SCCOHT). So far, no histogenetic origin of SCCOHT has been identified and accordingly, only little is known about tumor tissue characteristics of SCCOHT. Initial immunohistochemical analysis of the SCCOHT has postulated a germ cell-derived tumor [1] although electron microscopy evaluations of tumor specimen reported SCCOHT as an epithelial-like originating tumor [2]. Further analysis of SCCOHT tumor specimen suggested an inhomogeneous tumor entity which neither confirmed a germ cell-derived nor an epithelial cell-derived tumor origin [3–5]. The heterogeneity of these data may be explained in part by the limitations of biopsy material from patients. An appropriate cellular model for this tumor entity is represented by the BIN-67 cells [6]. Due to the unknown etiology, the SCCOHT which represents an aggressive form of ovarian tumors still remains with poor prognosis and no efficient therapy. Thus, the SCCOHT which is mostly accompanied by a paraendocrine hypercalcemia [2,7] preferably affects young women between ages of 13 to 35 with lethal outcome in a short period of time after diagnosis.

Potential therapeutic approaches to date are based predominantly on certain histological SCCOHT tissue examinations. The findings revealed that some areas of SCCOHT tumor stained positive for epithelial cell markers whereas the intermediate filament protein vimentin has been described in the majority of cells [3]. In addition, cell cycle analysis of several SCCOHT tumors by flow cytometry reported a broad distribution with 4.7% to 18% of S phase cells and 1.5% to 19.5% of G_2_/M phase cells [8], however, the histogenesis and further cell biological properties of the SCCOHT still remained poorly understood. Recent studies of a variety of SCCOHT tissue samples revealed a mutation in the *SMARCA4* gene as a potential marker for the SCCOHT [9–11].

Moreover, interaction of the tumor cells with adjacent populations within the tumor microenvironment including endothelial cells and mesenchymal stem cells support tumor vascularization and growth, however, such interaction alters the functionality and induces differentiation processes of the stem cells which can contribute to protect the tumorigenic target cells [12,13]. Consequently, reasonable approaches for the treatment of SCCOHT patients or a sufficient (chemo)therapeutic management are difficult and remain unclear. A recently developed cellular model of SCCOHT-1 cells derived from a primary culture of biopsy material after surgery of a 31-year-old patient with recurrent SCCOHT confirmed a cell type with epithelial/mesenchymal properties by partially expressing epithelial cytokeratins as well as the mesenchymal-type intermediate filament vimentin. Expression of surface markers in SCCOHT-1 includes CD15, CD29, CD44 and CD90 [14]. Based upon this cellular model of SCCOHT-1 cells, we examined in the present study cytotoxic effects of a variety of anti-tumor compounds in comparison to established human ovarian adenocarcinoma cell lines including NIH:OVCAR-3 and SK-OV-3 with known resistance to cisplatin [15]. The obtained *in vitro* effects in SCCOHT-1 cells with a focus on microtubule-stabilizing chemotherapeutics including epothilone B were investigated at the protein level to identify certain molecular effects and mechanisms. Moreover, epothilone B in combination with calcium was applied in NOD/scid mouse tumor xenografts to verify the *in vitro* therapeutic effects also *in vivo*. Our findings provide a more detailed understanding of potential compounds to target ovarian cancer cells exhibiting resistance to a variety of chemotherapeutics.

## Material and methods

### Cell culture

#### Primary human SCCOHT-1 cells

SCCOHT-1 cells were derived as a spontaneously permanent growing primary culture from a tumor biopsy after surgery of a 31-year-old patient with recurrent SCCOHT [14]. Informed written consent was obtained from the patient for the use of this material and the study has been approved by the Ethics Committee of Hannover Medical School, Project #3916 on June 15th, 2005. The SCCOHT-1 cells were cultured in RPMI 1640 supplemented with 10% (v/v) fetal calf serum, 2mM L-glutamine, 100U/ml penicillin and 100 μg/ml streptomycin. The tissue culture was performed at 37°C in a humidified atmosphere of 5% (v/v) CO_2_ and the medium was changed at intervals of 3 to 4 days. For subculture, the loosely attached cells were mechanically collected, centrifuged (320 g/6 min), and resuspended in growth medium at a cell viability of >95% as determined in a hemocytometer using the trypan blue exclusion test. The proliferation of SCCOHT-1 cells was measured in a fluorescence-based microtiter plate assay following transduction of SCCOHT-1 cells with a 3rd generation lentiviral SIN vector containing the eGFP gene as previously described for these cells [14].

The human SCCOHT cell line BIN-67 (kindly provided by Dr. Barbara Vanderhyden, University of Ottawa, Canada) was cultured with DMEM/F12 : DMEM medium (1:1) (Sigma Aldrich, St. Louis, MO) supplemented with 20% (v/v) fetal calf serum, 2mM L-glutamine, 100U/ml penicillin and 100 μg/ml streptomycin.

Human alveolar adenocarcinoma A549 cell line (kindly provided by Dr. Detlef Neumann, Hannover Medical School, Germany) were cultured with DMEM (Sigma) supplemented with 10% (v/v) fetal calf serum, 2mM L-glutamine, 100U/ml penicillin and 100 μg/ml streptomycin.

#### Human ovarian adenocarcinoma cell lines

Human NIH:OVCAR-3 ovarian cancer cells (ATCC® #HTB-161™) were commercially obtained in passage 76 (P76) from the Institute for Applied Cell Culture (IAZ), Munich, Germany. The SK-OV-3 ovarian cancer cells (ATCC® #HTB-77™) were commercially obtained in P25 from the ATCC, Manassas, VA, USA. These ovarian adenocarcinoma cell lines were originally established from the malignant ascites of a patient with progressive adenocarcinoma of the ovary, respectively. The cells were cultivated at about 1,750 cells/cm^2^ in RPMI 1640 supplemented with 10% (v/v) fetal calf serum, 2mM L-glutamine, 100U/ml penicillin and 100 μg/ml streptomycin. Subculture was performed by trypsin/EDTA (Biochrom GmbH, Berlin, Germany) treatment for 5 min at 37°C. For the experiments NIH:OVCAR-3 cells were used in P86 to P118 and SK-OV-3 cells were used in P37 to P39. For fluorescence measurement in an appropriate proliferation assay the NIH:OVCAR-3 as well as the SK-OV-3 cells have also been transduced with a 3rd generation lentiviral SIN vector containing the eGFP gene similar to SCCOHT-1 cells.

Authentication of SCCOHT-1, NIH:OVCAR-3, and SK-OV-3 cells was performed by short tandem repeat (STR) fragment analysis using the GenomeLab human STR primer set (Beckman Coulter Inc., Fullerton, CA, USA). PCR products were sequenced in a CEQ8000 Genetic Analysis System (Beckman Coulter) using the GenomeLab DNA size standard kit-600 (Beckman Coulter). The results of SCCOHT-1 were similar to the original SCCOHT patient cells cultured in our lab and the NIH:OVCAR-3 and SK-OV-3 cell lines results were similar to the STR database provided by the Deutsche Sammlung von Mikroorganismen und Zellkulturen (DSMZ, Braunschweig, Germany).

### Proliferation measurement by fluoroscan assay

The ovarian cancer cells were incubated with different concentrations for each of the chemotherapeutic compounds. The compounds and their concentrations used in the cell culture are:

carboplatin (320 μM; Carbomedac, Medac GmbH, Hamburg, Germany), cisplatin (320 μM; Bristol-Myers-Squibb), cyclophosphamide (1.28 mM; Cyclophostin, Pharmacia GmbH, Erlangen, Germany), cytarabine (320 μM; Ara C, Sigma Aldrich GmbH, München, Germany), 5’-fluorouracil (320 μM; Gry-Pharma GmbH, Kirchzarten, Germany), doxorubicin (1.28 μM; Sigma), methotrexate (320nM; Hexal AG, Holzkirchen, Germany), topotecan (320nM; Glaxo Smithkline GmbH&Co KG, Munich), taxol (160nM; Paclitaxel, Bristol-Myers-Squibb GmbH&Co KGaA, Munich), epothilone A (160nM; GBF Braunschweig, Germany), epothilone B (40nM; GBF Braunschweig, Germany), and ixabepilone (80 μM; Bristol-Myers-Squibb), respectively.

For fluorescence measurement the different eGFP-transduced ovarian cancer populations were seeded at 3,000 cells/well with standard culture medium (100 μL/well) in flat bottom 96-well plates (Nunc/ThermoFischer, Roskilde, Denmark) and incubated overnight to allow attachment. Thereafter, 100 μl of culture medium was added to the cells as control and in further wells 100 μl of culture medium with the maximal solvent concentration was added to the cells as solvent control, respectively. Moreover, 100 μl of the chemotherapeutic compounds were added to the cells and dosed in a 2-fold serial dilution. Each plate was applied with a cells-only control in culture medium and a maximal solvent concentration control, respectively (Table 1). The cell viability obtained with the appropriate chemotherapeutic compounds was then normalized to these controls on a plate by plate basis and a drug-dose–response analysis was performed for the different compounds in the 3 different ovarian cancer cell populations. Following incubation of the cells for 72 h, the medium was removed and the cells were lysed with 5% (w/v) SDS. Afterwards, the fluorescence intensities of GFP in the cell homogenate which corresponded to the appropriate cell number of ovarian cancer cells was measured at excitation 485 nm/emission 520 nm using the Fluoroscan Ascent Fl (Thermo Fisher Scientific). The resulting fluorescent signal was first normalized to the mean signal of the cells only wells to control for seeding variability and then to the mean signal of the solvent-only control.

**Table 1:** **Concentrations of chemotherapeutic compounds used in human ovarian cancer cells**

**Chemotherapeutic compound**	**Maximal solvent concentration [%]**	**Maximal chemotherapeutic compound concentration**		**IC50** [**M**]	
			**SCCOHT**-**1**	**SK**-**OV**-**3**	**NIH**:**OVCAR**-**3**
Cytarabine	1.6 × 10^0^ H_2_O	160 μM	8.1 × 10^-6^	1.0 × 10^-6^	1.1 × 10^-7^
Cisplatin	2.0 × 10^–2^ NaCl	160 μM	2.3 × 10^-5^	3.3 × 10^-6^	1.7 × 10^-6^
Carboplatin	5.0 × 10^–3^ NaCl	160 μM	7.9 × 10^-5^	8.8 × 10^-6^	7.0 × 10^-6^
Cyclophosphamide	7.5 × 10^–3^ NaCl	640 μM	1.0 × 10^-5^	2.3 × 10^-4^	1.0 × 10^-4^
Methotrexate	3.0 × 10^–5^ NaCl/ 2.0 × 10^–1^ PBS	160 nM	4.7 × 10^-9^	5.7 × 10^-9^	5.9 × 10^-9^
Topotecan	7.0 × 10^–3^ H_2_O/ 2.0 × 10^–1^ PBS	160 nM	3.6 × 10^-9^	1.8 × 10^-8^	5.0 × 10^-9^
Doxorubicin	3.0 × 10^–2^ H_2_O/ 2.0 × 10^–1^ PBS	640 nM	2.0 × 10^-8^	1.3 × 10^-7^	2.9 × 10^-8^
5‘-fluorouracil	4.0 × 10^–4^ NaCl/ 2.0 × 10^–1^ PBS	160 μM	1.9 × 10^-5^	3.5 × 10^-6^	1.1 × 10^-6^
Epothilone A	8.0 × 10^–3^ DMSO/ 2.0 × 10^–1^ PBS	80 nM	3.3 × 10^-9^	2.9 × 10^-9^	2.2 × 10^-9^
Epothilone B	2.0 × 10^–3^ DMSO/ 2.0 × 10^–1^ PBS	20 nM	1.5 × 10^-9^	2.9 × 10^-10^	9.8 × 10^-11^
Taxol	6.0 × 10^–4^ ethanlol/ 2.0 × 10^–1^ PBS	80 nM	2.2 × 10^-9^	2.4 × 10^-9^	1.4 × 10^-9^
Ixabepilone	2.0 × 10^–1^ ethanol	40 μM	1.1 × 10^-6^	1.6 × 10^-6^	9.8 × 10^-7^

The maximal chemotherapeutic compound concentration indicates the highest initial concentration on the cells in the well followed by 2-fold serial dilutions.

The IC50 values of the appropriate chemotherapeutic compounds were calculated from the drug-dose–response curves after normalization to the mean signal of the cells-only control and then to the mean signal of the solvent-only control.

### Cell cycle analysis

The cell cycle analysis was performed as described previously [16]. Briefly, 9.3 × 10^3^ cells/cm^2^ were seeded in culture plates (diameter 10 cm; Greiner Bio-one GmbH, Frickenhausen, Germany) overnight to allow attachment of the cells and adjustment to the culture conditions. Following incubation with 1 μM cisplatin, or 1 μM carboplatin, or 2nM epothilone B for 48 h, the cells were fixed in 70% (v/v) ice-cold ethanol at 4°C for 24 h. Thereafter, about 5 × 10^5^ fixed cells were stained with CyStain DNA 2 step kit (Partec GmbH, Münster, Germany) and filtered through a 50 μm filter. The samples were then analyzed in a Galaxy flow cytometer (Partec) using the MultiCycle cell cycle software (Phoenix Flow Systems Inc., San Diego, CA).

### Immunoblot analysis

For immunoblot analysis, untreated and chemotherapeutic agents-stimulated SCCOHT-1^GFP^, NIH:OVCAR-3^GFP^ and SK-OV-3^GFP^ cells were washed three times in ice-cold PBS and lysed in a reswelling buffer containing 8 M urea (Carl Roth GmbH Co KG, Karlsruhe, Germany), 1% CHAPS (3-[(3-Cholamidopropyl)dimethylammonio]-1-propanesulfonate) (Carl Roth GmbH Co KG), 0.5% (v/v) Pharmalyte 3 –10 (GE Healthcare Europe GmbH, Freiburg, Germany), 0.002% (w/v) bromophenol blue (SERVA Electrophoresis GmbH, Heidelberg, Germany) and freshly prepared 0.4% (w/v) DTT (Dithiothreitol) (Carl Roth GmbH Co KG). Protein concentration was adjusted using the colorimetric BCA-assay (ThermoScientic, Rockford, IL, USA), subjected to SDS-polyacrylamide gel electrophoresis and transferred to a hybond-C extra nitrocellulose membrane (GE Healthcare). The membranes were blocked with PBS containing 5% FCS and 0.05% Tween-20 (PBS/Tween). After washing four times with PBS/Tween, the membranes were incubated with the primary antibodies (monoclonal anti-BRG-1 (dilution 1:1,000; ab110641; Abcam plc, Cambridge, UK); polyclonal anti-p53^[pSer15]^ (dilution 1:1,000; Cell Signaling Technology, Beverly, MA, USA); polyclonal anti-p53 (dilution 1:1,000; Cell Signaling Technology); monoclonal anti-HSP27^[pSer82]^ (dilution 1:200; clone 5B9, Enzo GmbH, Lörrach, Germany); monoclonal anti-tubulinβ3 (dilution 1:500; clone TU-20, Novus Biologicals Ltd., Cambridge, UK); monoclonal anti-β-actin (dilution 1:5,000; clone AC-15; Sigma-Aldrich) and monoclonal anti-GAPDH (dilution 1:200; clone AC-15 (Santa Cruz Biotechnology, Santa Cruz, CA, USA)) overnight at 4°C. Thereafter, the membranes were washed four times with PBS/Tween and incubated with the appropriate horseradish peroxidase-conjugated anti-mouse IgG (dilution 1:5,000) or anti-rabbit IgG (dilution 1:10,000) secondary antibody, respectively, (all from GE Healthcare, Freiburg, Germany) for 1 h/room temperature. The membranes were washed with PBS/Tween and visualized by autoradiography using the ECL-detection kit (GE Healthcare). Quantification of the blots was performed by densitometry scanning using the Image J program.

### *In vivo* experiments

Animal research using NOD/scid mice was carried out by following internationally recognized guidelines on animal welfare and has been approved by the institutional licensing committee ref. #33.14-42502-04-12/0814 on June 22nd, 2012.

About 1 x 10^6^ GFP-labeled SCCOHT-1 cells previously cultured in serum-free HybridoMed DIF 1000 medium to avoid non-specific serum effects were injected subcutaneously into 5 to 6 weeks old female NOD/scid mice, respectively. After about 18 days post injection, all mice with SCCOHT-1^GFP^ cells had developed subcutaneous tumors. A therapeutic approach of the tumors was first tested with a daily subcutaneous injection of only 200 μl epithilone B (10 μM Epo B) at the tumor site for 2 days. To test possible synergistic effects of calcium and epothilone B in a further set of experiments, tumor-carrying mice were divided into 3 treatment groups. The first group represented the control tumor group with 5 animals and was injected subcutaneously with 200 μl of 0.9% NaCl at the tumor site every day. The second group with 5 animals was injected subcutaneously with 200 μl Ca^2+^ (5 mM) in 0.9% NaCl at the tumor site every day. The third group of 5 animals with tumor-carrying mice was injected subcutaneously with 200 μl Ca^2+^ (5 mM) together with 10 μM Epo B in 0.9% NaCl at the tumor site every day. The tumor length (L) and width (W) in each animal was measured on a daily basis and the resulting tumor size was calculated as ½ L × W^2^ where L is the longer of the 2 measurements according to the calculation of ellipsoid tumor forms [17]. The treatment was started at an initial tumor size of approximately 2 to 3 mm^3^.

At the end of the experiments, the animals were sacrificed by CO_2_ anesthesia and cervical dislocation. Following UV light examination for the detection of GFP positive tissue, the tumors were dissected whereby tumor weight and the corresponding animal weight were determined.

For calcium measurements cardial blood was taken from the tumor-carrying NOD/scid mice after therapy and serum was prepared and analyzed for Ca^2+^ concentration using the Calcium Gen.2 reagent kit (Roche Diagnostics, Mannheim, Germany). The Ca^2+^ test is based on a color reaction with the chromophor 5-nitro-5’-methyl-1,2-bis(o-aminophenoxy)ethane- N,N,N',N'-tetraacetic acid (NM-BAPTA) according to the manufacturer’s instruction (Roche Diagnostics).

## Results

**Figure 1:**
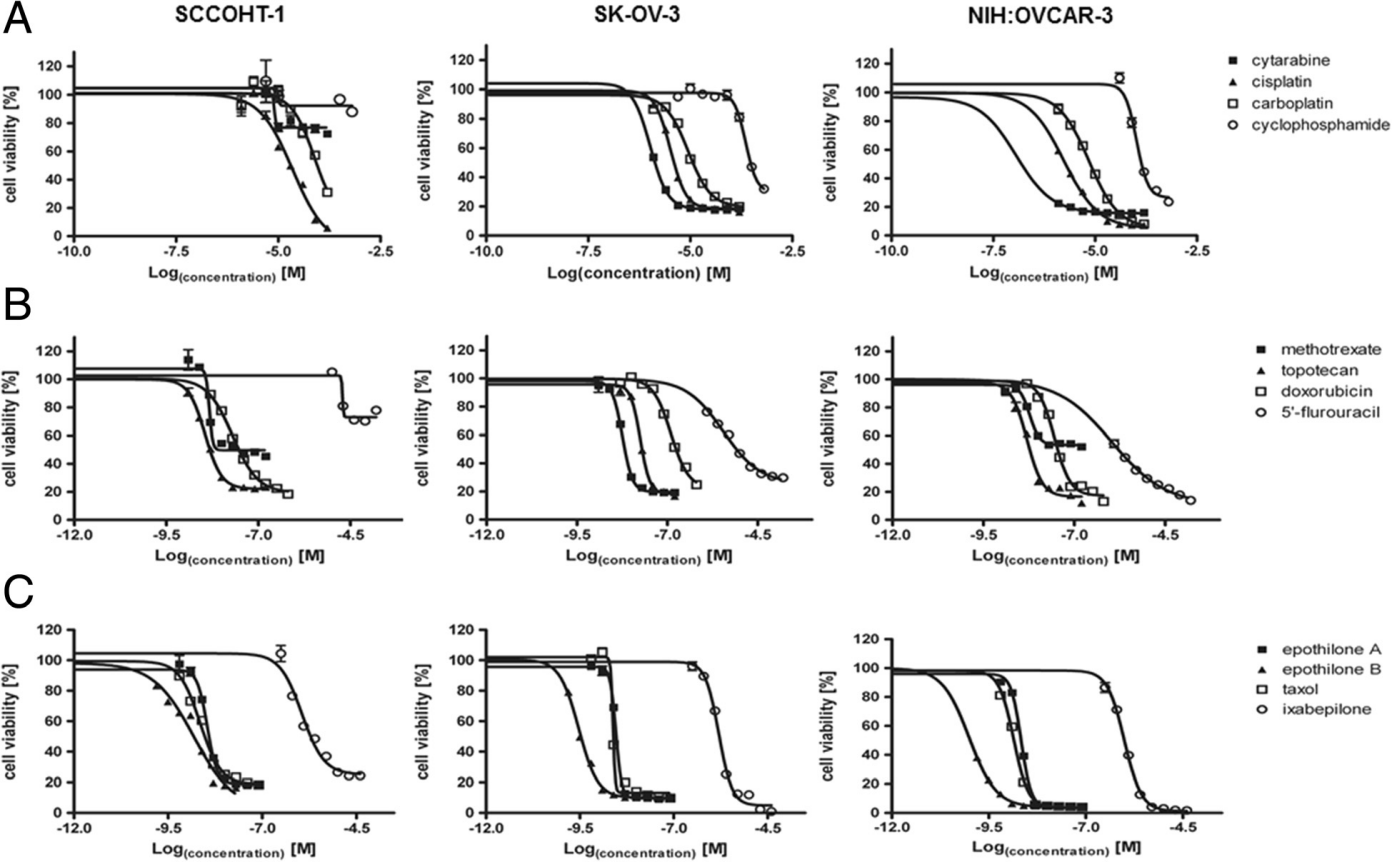
**Sensitivity of human ovarian cancer cells for chemotherapeutic compounds (A-C).** SCCOHT-1^GFP^ cells, NIH:OVCAR-3^GFP^ and SK-OV-3^GFP^ cells were incubated with different concentrations of chemotherapeutic compounds for 72 h, respectively, and the proliferative capacity was measured by the fluoroscan assay. Analysis of a drug-dose–response to define IC50 concentrations for all compounds was performed using GraphPad Prism-6. For calculation of the drug-dose–response curves, the data were normalized to the cells-only control in culture medium and to the maximal solvent concentration control of the corresponding compound, respectively.

To date, little if any successful chemotherapy is available for the poor prognosis SCCOHT and therefore, *in vitro* testing was performed using a recently developed cellular model of human SCCOHT-1 cells derived from a recurrent small cell ovarian carcinoma of the hypercalcemic type [14]. The proliferative capacity of SCCOHT-1 cells was tested in comparison to NIH:OVCAR-3 and SK-OV-3 ovarian carcinoma cells in a fluorescence-based assay of GFP-labeled cells following treatment with different chemotherapeutic compounds for 72 h (Figure 1A-C). DMSO as an initial solvent for certain compounds was diluted to less than 0.1% (v/v) in the final concentration whereby incubation of the cells with even 0.2% (v/v) DMSO displayed no detectable effects as compared to control cells without DMSO reaching a proliferation rate of 104.3% ± 9.2% (n = 6) after 72 h.

Incubation of the cells with cisplatin revealed an IC50 of 2.3 × 10^−5^ M in SCCOHT-1^GFP^ cells and 3.3 × 10^−6^ M and 1.7x10^−6^ M in SK-OV-3^GFP^ and NIH:OVCAR-3^GFP^ cells, respectively (Figure 1A). Likewise, little effects on the proliferation of these 3 ovarian cancer populations were observed after exposure to carboplatin for up to 72 h (Figure 1A, Table 1). Only a marginal growth inhibition of SCCOHT-1^GFP^, SK-OV-3^GFP^ and NIH:OVCAR-3^GFP^ cells was also detectable following incubation of the cells with cytarabine and even less with cyclophosphamide (Figure 1A, Table 1). Similarly, 5’-fluoruracil displayed only little effects on the proliferation of the 3 ovarian cancer cell types (Figure 1B, Table 1). In contrast, exposure to doxorubicin, topotecan and methotrexate was associated with a significantly elevated inhibiton of the proliferative capacity in SCCOHT-1^GFP^, SK-OV-3^GFP^ and NIH:OVCAR-3^GFP^ cells, respectively (Figure 1B, Table 1).

Mitotic inhibitors which stabilize the microtubules including taxol and epothilones exhibited different anti-proliferative effects. Thus, taxol revealed a growth reduction of in SCCOHT-1^GFP^ with a IC50 of 2.2nM which was enhanced in NIH:OVCAR-3 cells displaying an IC50 of 1.4nM but less pronounced in SK-OV-3^GFP^ cells with an IC50 of 2.4nM (Figure 1C, Table 1). Whereas epothilone A demonstrated a slightly reduced sensitivity as compared to taxol, treatment of the 3 different ovarian cancer cell populations to epothilone B revealed the highest growth inhibition tested in this study displaying an IC50 of 1.5nM for SCCOHT-1^GFP^, 0.3nM for SK-OV-3^GFP^, and 0.098nM for NIH:OVCAR-3^GFP^ cells (Figure 1C, Table 1). In contrast, only a low responsiveness of the cells was observed to ixabepilone with IC50 value in the micromolar range (Figure 1C, Table 1).

Together, these findings demonstrated differences in the chemotherapeutic sensitivity of these 3 ovarian cancer populations. Moreover, topotecan, methotrexate, taxol and epothilone B appeared as the most potent chemotherapeutic compounds for SCCOHT-1^GFP^ cells *in vitro* with the highest potency for epothilone B.

**Figure 2:**
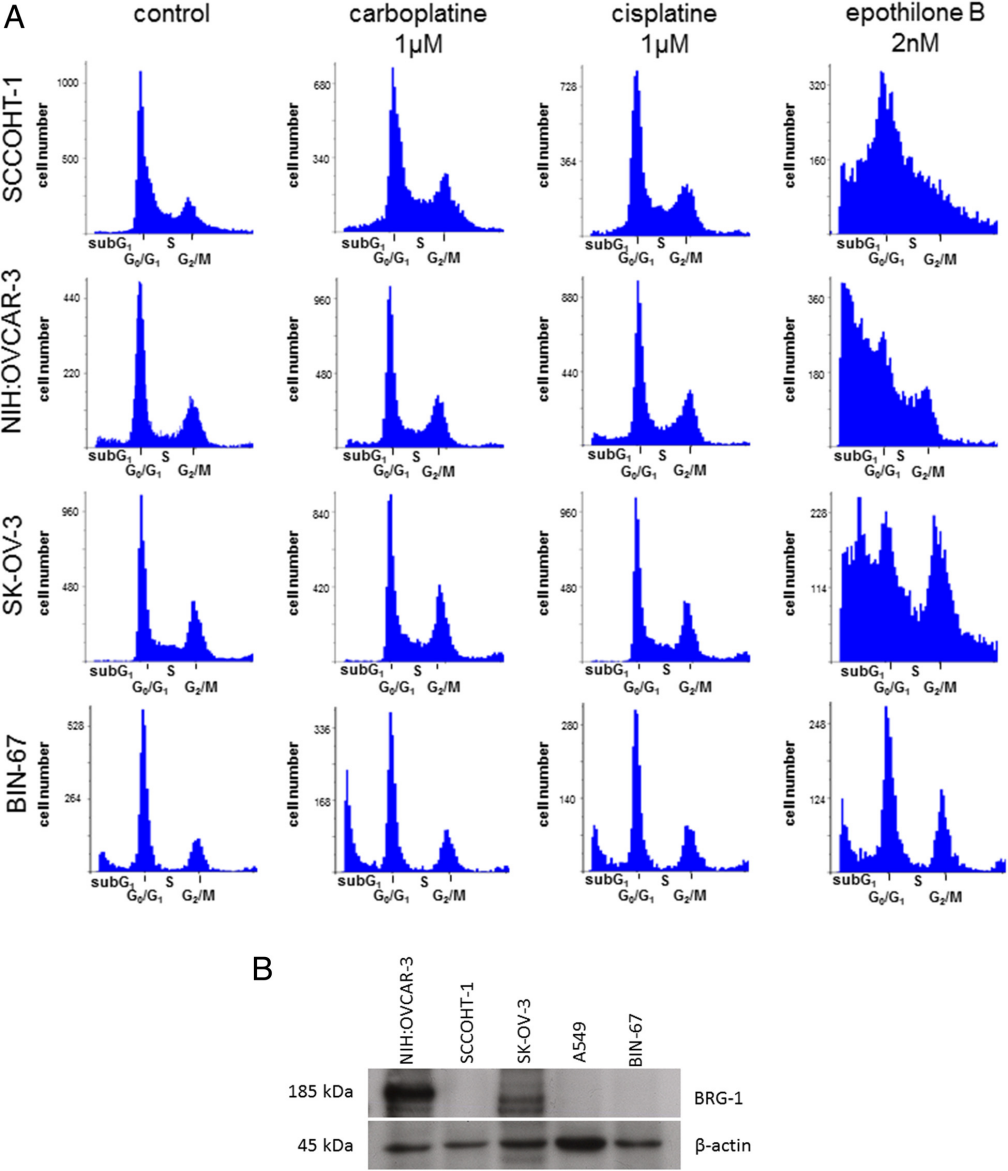
**Cell cycle analysis of ovarian cancer cells after chemotherapeutic compound application (A) and SMARCA4/BRG-1 protein expression in human ovarian cancer cells (B).** Cell cycle analysis **(A)** was performed by flow cytometry in SCCOHT-1^GFP^, NIH:OVCAR-3^GFP^, SK-OV-3^GFP^ and BIN-67 cells after treatment with 1 μM cisplatin, or 1 μM carboplatin, or 2 nM epothilone B for 48 h, respectively. Western blot analysis **(B)** of BRG-1 protein was performed in steady state of two ovarian carcinoma cell lines (NIH:OVCAR-3 and SK-OV-3) as compared to SCCOHT-derived cells (SCCOHT-1 and BIN-67). The absence of BRG-1 in human alveolar adenocarcinoma A549 cells was used as a control and expression of β-actin served as a loading control.

Further analysis was performed to test the effects of epothilone B on the cell cycle progression of the ovarian cancer cells in comparison to cisplatin or carboplatin which are frequently used in a combination with etoposide or taxol, respectively, for treatment of the SCCOHT [18,19]. Flow cytometric cell cycle analysis of logarithmically-growing SCCOHT-1^GFP^ cells revealed a distribution of continuously proliferating cells with about 68% in G_0_/G_1_ phase, 9% in S phase and 23% in the mitotic G_2_/M phase as evaluated by the MultiCycle cell cycle software (Figure 2A). A similar cell cycle distribution of continuously proliferating cells was observed following incubation of SCCOHT-1^GFP^ cells with either 1 μM cisplatin or 1 μM carboplatin for 48 h. In contrast, treatment of SCCOHT-1^GFP^ cells with a 500-fold reduced concentration of 2 nM epothilone B for 48 h was associated with G_0_/G_1_ cell cycle arrest and a significant accumulation of dead cells in the subG_1_ phase (Figure 2A). Likewise, the platin-resistent ovarian cancer cell lines NIH:OVCAR-3 and SK-OV-3 demonstrated a paralleled cell cycle pattern following exposure to 1 μM cisplatin or 1 μM carboplatin or 2 nM epothilone B for 48 h whereby SK-OV-3 also displayed an accumulation in G_2_/M upon epothilone B exposure (Figure 2A). The SCCOHT-derived cell line BIN-67 demonstrated platin-compound resistance although some subG1 accumulation was detectable following treatment with 1 μM carboplatine for 48 h. Moreover, incubation of BIN-67 cells to 2 nM epothilone B revealed an accumulation in G_2_/M phase (Figure 2A). These findings substantiated the unresponsiveness of BIN-67 and SCCOHT-1 cells as well as NIH:OVCAR-3 and SK-OV-3 cells to platin-based compounds. Moreover, growth inhibitory effects of epothilone B associated with significant cellular damage and cell death were confirmed in the ovarian cancer lines except for BIN-67 cells with a markedly reduced sensitivity.

Differences between ovarian cancer cells and cells derived from SCCOHT have been previously reported by a mutation in the *SMARCA4* gene as a potential marker for the SCCOHT [9–11]. Western blot analysis of BRG-1 as the protein product of the *SMARCA4* gene revealed a pronounced expression in the NIH:OVCAR-3 and SK-OV-3 ovarian cancer cells, however, little if any BRG-1 protein was detectable in SCCOHT-1 cells (Figure 2B). Likewise, BRG-1 was absent in human alveolar adenocarcinoma A549 cells and in the BIN-67 cell line as previously reported [11] (Figure 2B), suggesting also a *SMARCA4* defect in SCCOHT-1 cells. Detection of β-actin expression was used as a loading control (Figure 2B).

**Figure 3:**
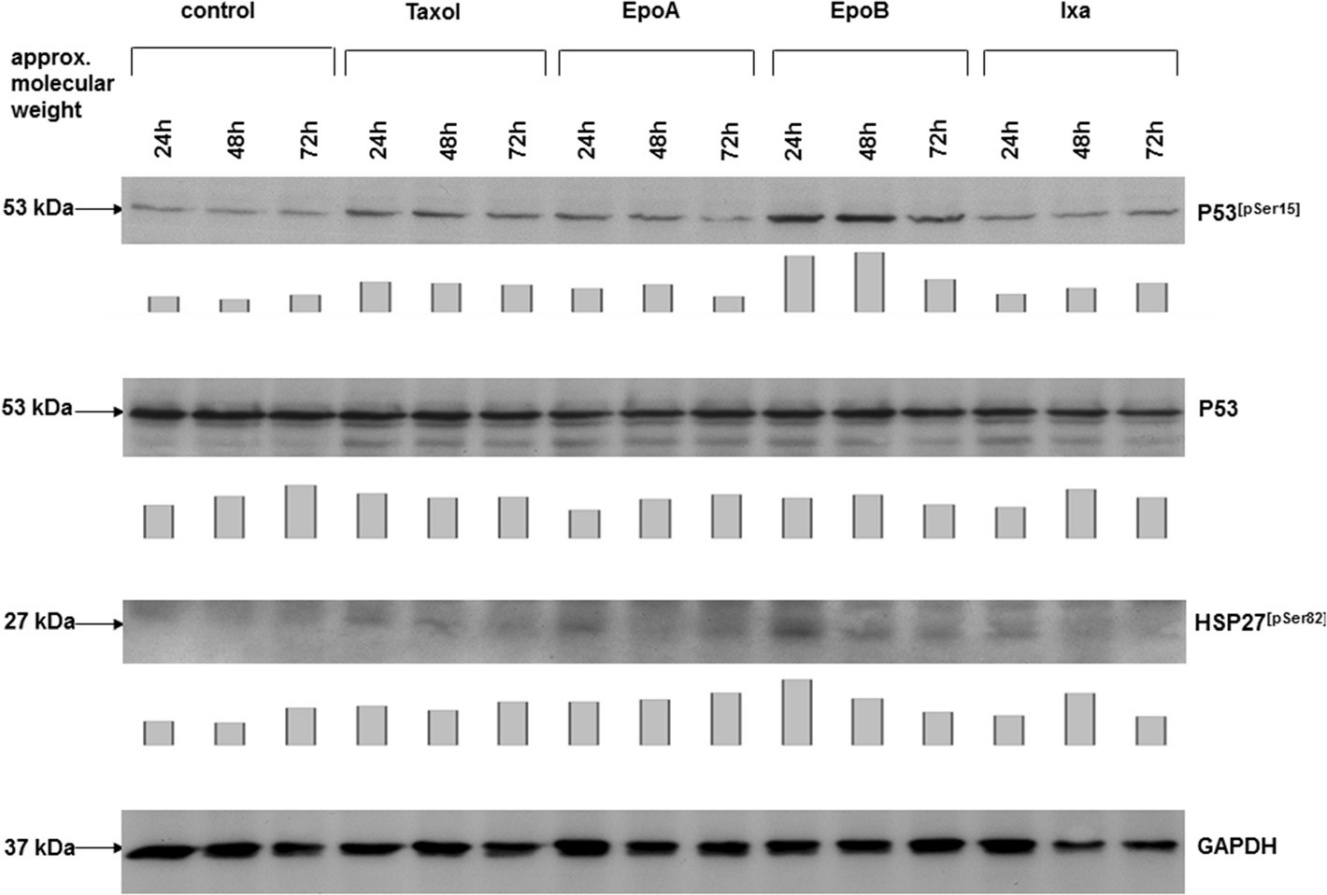
**Western blot analysis was performed in SCCOHT**-**1 cells**, **treated with 4 nM of either taxol**, **epothilone A (EpoA), epothilone B (EpoB), or ixabepilone (Ixa) for 24 h**, **48 h**, **and 72 h**, **respectively**. Protein aliquots of the corresponding cell homogenates were analysed for the expression of p53, phosphorylated p53 at serine-15, and phosphorylated heat shock protein (HSP27) at serine-82. The unaltered expression of GAPDH served as a control for equal loading. Quantification of the blots by densitometry scanning was normalized against the appropriate GAPDH expression and the relative expression levels were documented as bar diagram below the corresponding Western blots.

Whereas cellular and DNA damage activate a cascade of repair mechanisms involving p53 and distinct phosphorylation processes of this tumor suppressor protein, the different responses of SCCOHT-1 cells observed with taxol and certain epothilones, particularly epothilone B and ixabepilone, were evaluated by Western blot analysis. Thus, only marginal differences were observed for the protein level of p53 expression in SCCOHT-1 cells following treatment with either taxol, epithilone A, epithilone B, or ixabepilone. However, there was a significantly enhanced detection of phosphorylated p53 at serine15 (p53^[pSer15]^) particularly between 24 h to 48 h after epothilone B treatment (Figure 3). Likewise, an elevated phosphorylation of the heat shock protein HSP27 at serine 82 (HSP27^[pSer82]^) could be detected within 24 h to 48 h of epothilone B exposure together with unchanged control expression of GAPDH (Figure 3). Quantification by densitometry scanning also revealed an elevated expression of p53^[pSer15]^ in taxol-stimulated SCCOHT-1 cells, however, these levels remained significantly lower as compared to those observed after epithilone B treatment.

**Figure 4:**
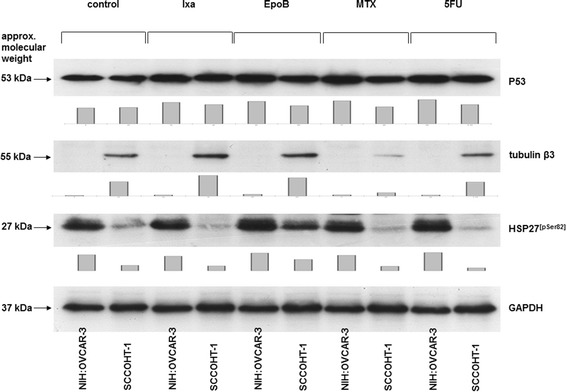
**Comparison of protein expression in SCCOHT-1 and NIH:OVACAR-3 cells.** Western blot analysis was performed in SCCOHT-1^GFP^ and NIH:OVCAR-3^GFP^, to compare the protein expression levels of p53, tubulinβ3, and phosphorylated heat shock protein (HSP27) at serine-82 after a 24 h incubation of the 2 cell types with 2 nM of ixabepilone (Ixa), epothilone B (EpoB), methotrexate (MTX), and 5’-fluorouracil (5FU), respectively. The unaltered expression of GAPDH served as a control for equal loading, respectively. Quantification of the blots by densitometry scanning was normalized against the appropriate GAPDH expression and the relative expression levels were documented as bar diagram below the corresponding Western blots.

A comparison of distinct chemotherapeutic effects between SCCOHT-1 and NIH:OVCAR-3 cells revealed little if any change in p53 protein expression of the investigated compounds, whereby densitometric analysis revealed slightly induced p53 levels in the compound-treated NIH:OVCAR-3 as compared to SCCOHT-1 cells (Figure 4). A significant difference, however, was observed for the tubulinβ3 protein level which was constitutively expressed already in untreated SCCOHT-1 cells and decreased after methotrexate treatment in contrast to undetectable tubulinβ3 protein in NIH:OVCAR-3 cells (Figure 4). Conversely, HSP27^[pSer82]^ was significantly higher and unaltered expressed in NIH:OVCAR-3 cells as in SCCOHT-1 cells whereby incubation with epothilone B for 24 h was associated with an increase of HSP27^[pSer82]^ protein levels in SCCOHT-1 cells (Figure 4). SK-OV-3 cells are reported as p53 defective and a similar expression pattern as compared to NIH:OVCAR-3 cells was also observed for HSP27^[pSer82]^ and undetectable tubulinβ3 (data not shown).

**Figure 5:**
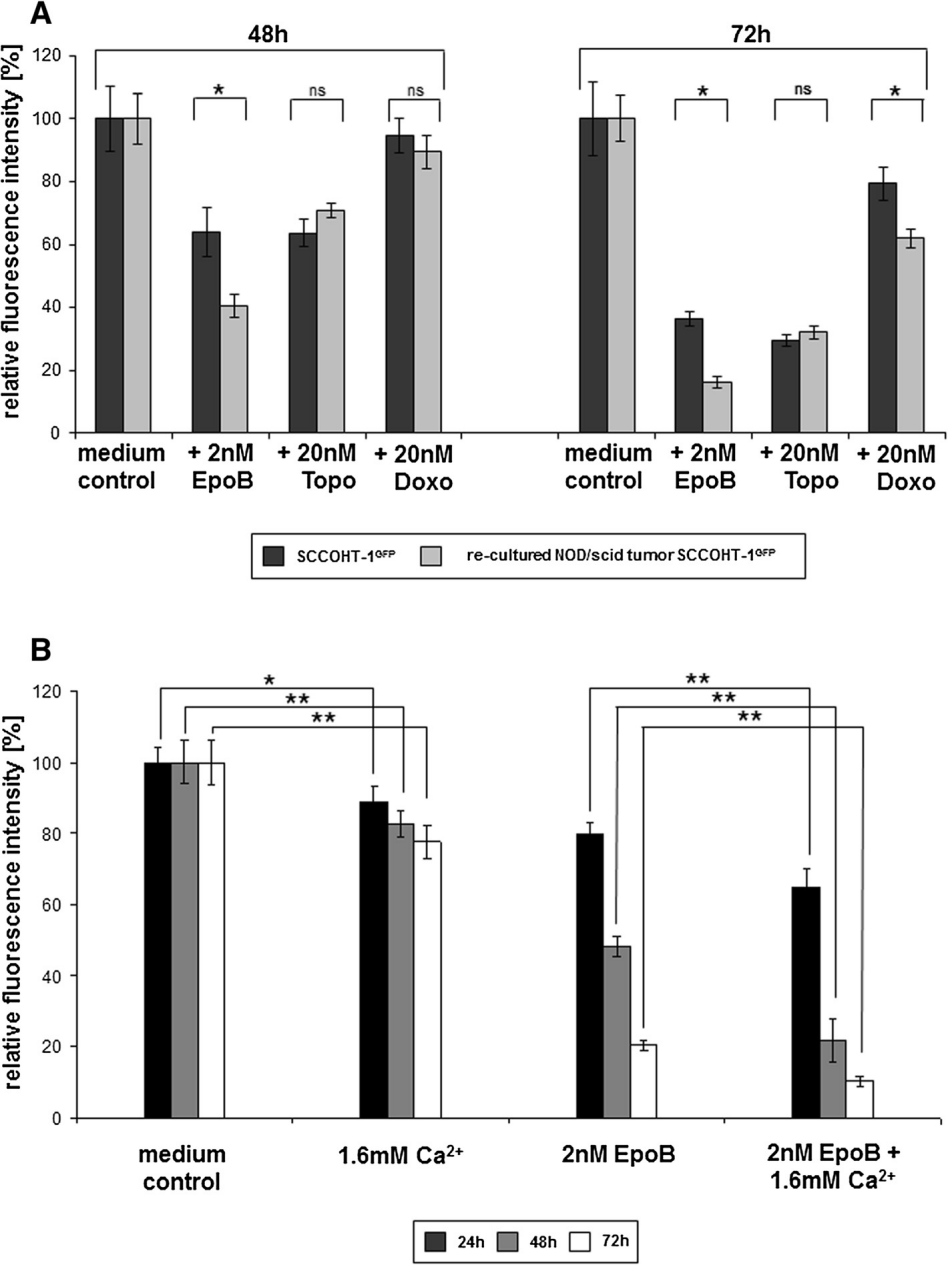
**Chemotherapeutic sensitivity of re-cultured SCCOHT**-**1** ^**GFP**^ **cells from xenograft tumors (A) and sensitivity of SCCOHT-1 cells to Ca** ^**2+**^ **and Epo B (B).** SCCOHT-1^GFP^ cells and re**-**cultured cells obtained from a SCCOHT-1^GFP^-induced tumor in NOD/scid mice **(A)** were incubated with 2 nM epothilone B (EpoB), 20 nM topotecan (Topo), and 20 nM doxorubicin (Doxo) for 48 h and 72 h, respectively, and the proliferative capacity was measured by the fluoroscan assay. Fluorescence data of the non-treated control cells were calculated as 100% of relative fluorescence intensity. Data represent the mean ± s.d. (n = 10 for each control; n = 9 for each chemotherapeutic compounds). Statistical analysis between the SCCOHT-1^GFP^ cells and re-cultured NOD/scid tumor SCCOHT-1^GFP^ cells after treatment with the chemotherapeutics was calculated by 2-way ANOVA following Tukey’s multiple comparison test (ns = not significant; *= significant (p < 0.0001)). SCCOHT-1^GFP^ cells were incubated with either 1.6 mM CaCl_2_ (1.6 mM Ca^2+^), 2 nM epothilone B (EpoB), or a combination of 2 nM EpoB + 1.6 mM Ca^2+^ in a fluoroscan assay for 24 h up to 72 h **(B)**, respectively. Fluorescence data of the non-treated control cells were calculated as 100% of relative fluorescence intensity. Data represent the mean ± s.d. (n = 10). Statistical analysis was conducted between SCCOHT-1^GFP^ cells in control medium and after exposure to 1.6 mM Ca^2+^ as well as between SCCOHT-1^GFP^ cells in the presence of 2 nM Epo B and SCCOHT-1^GFP^ cells after exposure to 2 nM EpoB + 1.6 mM Ca^2+^ by unpaired Student's t-test (*P < 0.0001; **P < 0.00001).

To further address the question whether these *in vitro* effects of epothilone B on SCCOHT-1 cells may also be effective *in vivo*, subcutaneous tumors were induced in NOD/scid mouse xenografts. Injection of 10^6^ GFP-labeled SCCOHT-1 cells resulted in a detectable tumor development within 2–3 weeks. First, NOD/scid mouse tumors were dissected and re-cultured to investigate whether the cells obtained from the re-cultured tumors maintain a similar chemotherapeutic sensitivity observed during previous *in vitro* culture of SCCOHT-1^GFP^ cells. Indeed, incubation of the NOD/scid mouse tumors re-cultured cells demonstrated a significantly increased sensitivity for epothilone B after 48 h and 72 h, respectively, whereas the responsiveness to topotecan-treated cells remained unaltered (Figure 5A). Conversely, a higher sensitivity was observed for doxorubicin which became significant after 72 h as statistically analyzed by 2-way ANOVA (Figure 5A). According to the hypercalcemia which accompanies this tumor disease, additional questions were addressed whether exogenous calcium affects SCCOHT-1 tumor cell growth *in vitro* together with epothilone B. Indeed, addition of 1.6 mM CaCl_2_ was associated with a continuously reduced proliferation of SCCOHT-1 cells in the fluoroscan assay by about 22.4% ± 4.6% (n = 10) after 72 h (Figure 5B). However, Ca^2+^ exhibited at least additive effects together with epothilone B and further diminished the epothilone B-mediated progressive growth reduction of SCCOHT-1 cells. Thus, the effects of epothilone B which reduced the SCCOHT-1 cell growth by 20.3% ± 3.6% (n = 10) after 24 h was further enhanced to 35.0% ± 5.3% (n = 10) together with Ca^2+^. Similar synergistic effects of Ca^2+^ together with an epothilone B-conferred growth inhibition of SCCOHT-1 cells were observed after 48 h and 72 h, respectively (Figure 5B).

**Figure 6:**
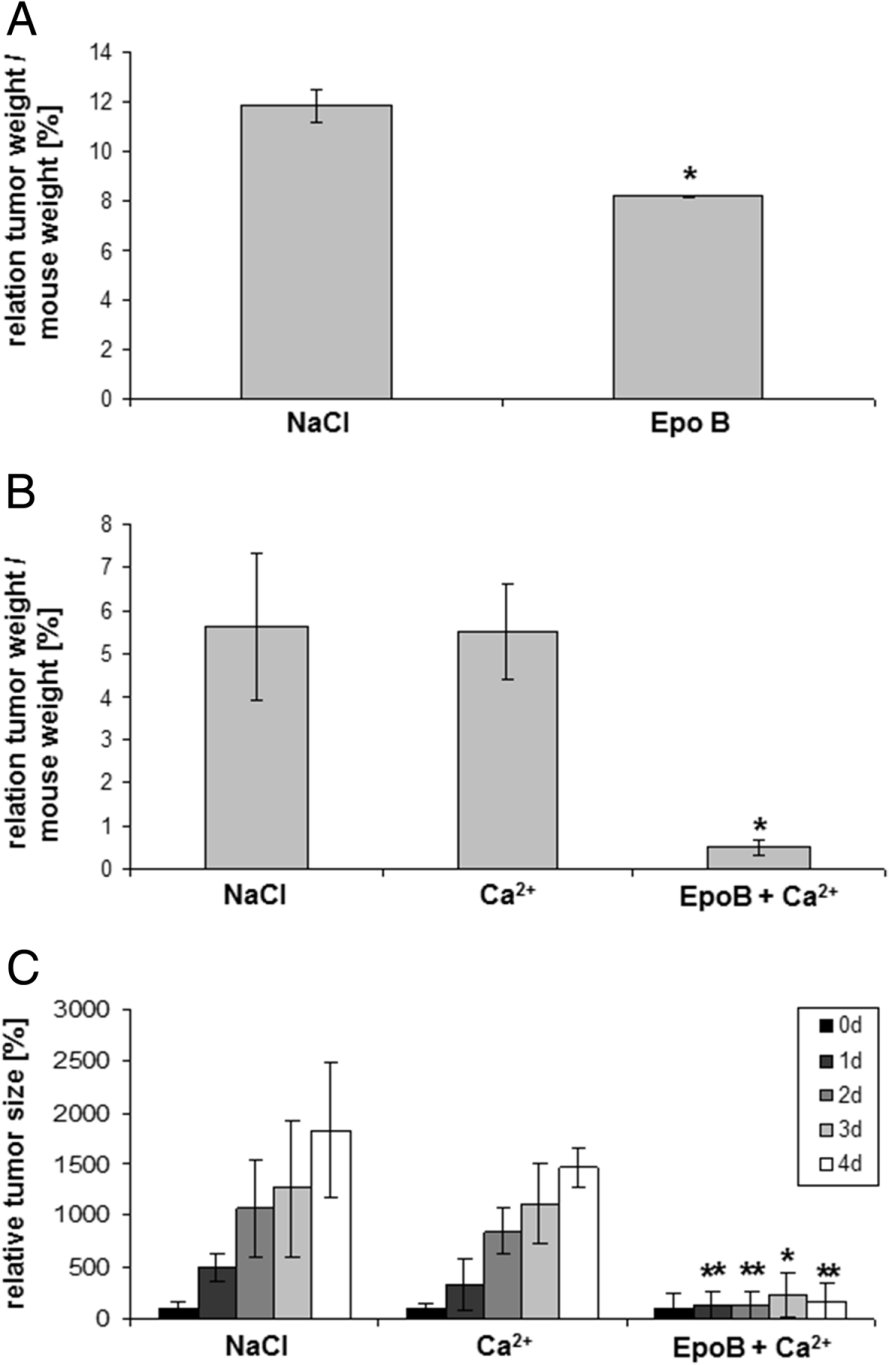
***In vivo*** **effects of different therapeutic approaches were examined in NOD/** **scid mice by evaluation of the tumor size each day in the course of the therapy and by calculation of the relation of tumor weight/** **mouse weight after tumor dissection at the end of the experiment**. Subcutaneous tumors were induced in NOD/scid mice within 18d following injection of about 10^6^ SCCOHT-1^GFP^ cells. Thereafter, different therapeutic approaches were applied by injection of 200 μl of either 0.9% NaCl, or 5 mM CaCl_2_, or 10 μM epothilone B (EpoB), or 5 mM CaCl_2_ + 10 μM EpoB each day. **A**. The relation of tumor weight / mouse weight was calculated after 2d of subsequent injections for control tumors (NaCl) (n = 2) and EpoB-treated tumors (n = 2). **B**. The relation of tumor weight / mouse weight was calculated after 4d of subsequent injections as the mean ± s.d. for control tumors (NaCl) (n = 5), Ca^2+^-treated tumors (n = 5), and Ca^2+^ + EpoB-treated tumors (n = 4). Statistical analysis was conducted by 1-way ANOVA test (*p = 0.027). **C**. The tumor size was evaluated each day at 4 consecutive days of subsequent injections as the mean ± s.d. for control tumors (NaCl) (n = 5), Ca^2+^-treated tumors (n = 5), and Ca^2+^ + EpoB-treated tumors (n = 4). Statistical analysis was calculated by unpaired Student's t-test between Ca^2+^-treated tumors and the NaCl-treated tumor sizes as well as between Ca^2+^ + EpoB-treated tumors and the NaCl-treated tumor sizes at the corresponding time points, respectively (*P < 0.05; **P < 0.01).

Based upon these results, further NOD/scid mouse tumors were examined for a successful therapeutic approach by a daily injection of epothilone B at the tumor site. Testing various concentrations revealed detectable effects with 10 μM epothilone B already after 48 h (=2 treatments) with a tumor size of 2.1 ± 0.2 cm^3^ as compared to 2.5 ± 0.1 cm^3^ in NaCl-treated control tumors and a relation of tumor weight to mouse weight of 8.2 ± 0.1 in the epothilone B-treated tumor mice as compared to 11.8 ± 0.6 in the NaCl-treated control tumors (Figure 6A). Although in this first therapeutic approach, the mice had to be sacrificed after 2 days for ethical reasons due to the tumor size, these data demonstrated already a reduction in tumor size by about 16% and a reduction in the relation of tumor weight to mouse weight by about 30% after solely epothilone B treatment.

Since addition of exogenous Ca^2+^ supported the growth-inhibitory effects of epothilone B *in vitro*, a further therapeutic approach was tested *in vivo* whereby 200 μl of 5 mM CaCl_2_ were injected at the tumor site and compared to the effects of a co-injection of 5 mM CaCl_2_ + 10 μM epothilone B. Although Ca^2+^ alone demonstrated little if any effects on the *in vivo* tumor growth, the combined treatment of Ca^2+^ + epothilone B was associated with a significant reduction by about 90% in the relation of tumor weight to mouse weight after tumor dissection of the sacrificed mice (Figure 6B). Thus, a therapeutic approach with CaCl_2_ + epothilone B reached a relation of tumor weight to mouse weight of 0.5 ± 0.2 (n = 3) after 4d, however, Ca^2+^ alone and the NaCl control injection reached a relation of tumor weight to mouse weight of 5.5 ± 1.0 (n = 4) and 5.6 ± 1.7 (n = 4), respectively. Statistical analysis by 1-way ANOVA revealed a significant tumor reduction (p = 0.027) (Figure 6B). Daily consecutive measurements and corresponding calculations of the tumor size normalized to the size at the beginning of the therapy (day 0) confirmed a more than 90% reduced tumor size in CaCl_2_ + epothilone B-treated mice. A consecutive NaCl control treatment and solely Ca^2+^ treatment revealed a rapidly growing and continuously increasing tumor size to 1827% ± 656% (n = 5) and 1472% ± 196% (n = 5) after 4d (Figure 6C). In contrast, consecutive treatment with CaCl_2_ + epothilone B for 4d was associated with an attenuation of tumor growth reaching an average tumor size of 165% ± 186% (n = 4). These findings suggested that a significantly reduced tumor growth of the SCCOHT *in vivo* by epothilone B treatment could be further enhanced by the addition of exogenous Ca^2+^ to the epothilone B therapy. This therapeutic effect of Ca^2+^/epothilone B was also accompanied by an abolished hypercalcemia in the mice. Whereas NaCl-treated control tumor-carrying mice and Ca^2+^-treated mice exhibited a hypercalcemia with average calcium levels in the blood serum of 3.11 ± 0.75 mmol/L (n = 3) and 3.20 ± 0.40 mmol/L (n = 4), respectively, the combined treatment of Ca^2+^/epothilone B demonstrated normal calcium serum levels of 2.16 ± 0.53 mmol/L (n = 3).

## Discussion

SCCOHT represents an aggressive female tumor with poor prognosis and previous work has suggested a multi-modality treatment for the SCCOHT including surgery and a subsequent chemotherapy consisting of cisplatin- and etoposide-based or carboplatin- and taxane-based components followed by a radiotherapy [18,19]. Despite this multi-modality approach, however, the level of tumor relapses remains high and only very few patients survived for more than two years [20–23]. Thus, the data obtained in this study demonstrated a certain resistance of SCCOHT-1 cells to a cisplatin- or carboplatin-based chemotherapy since both compounds were ineffective to decrease the proliferative capacity *in vitro*. Resistance to the platin chemotherapeutics has also been confirmed for the NIH:OVCAR-3 and SK-OV-3 ovarian cancer cells. Moreover, the continuous and unaltered cell cycle progression of SCCOHT-1 cells in the presence of both platin compounds is further questioning the effectiveness of these drugs in patients with SCCOHT. Our findings are also supported by studies in BIN-67 cells, displaying a resistance to platinum and other standard chemotherapeutic agents [24]. In contrast, microtubule-stabilizing compounds such as taxol and more importantly, epothilone B demonstrated significant anti-proliferative effects in SCCOHT-1 as well as in NIH:OVCAR-3 and SK-OV-3 cells *in vitro*. The growth-inhibitory effects of epothilone B were associated with an activation of the cellular and DNA damage response machinery including enhanced detection of HSP27^[pSer82]^ and p53^[pSer15]^, respectively, followed by increased cell death as determined via subG_1_ phase cell cycle accumulation in SCCOHT-1 cells. HSP27 phosphorylation can be mediated by PKD upon cellular stress and plays an important role in cellular protection [25]. Moreover, DNA damage response and cell cycle arrest is relayed via p53 phosphorylation including accumulation of p53^[pSer15]^ [26]. Treatment of human A2780 ovarian cancer cells with taxol has been reported with p53 phosphorylation at serin 20 [27] whereby taxol and epothilone B may confer signals for different phosphorylation sites at p53. In addition, epothilone-mediated cytotoxicity is relayed via different forms of p53 [28].

These significant cytotoxic effects of epothilone B in SCCOHT-1 cells *in vitro* could also be substantiated in a therapeutic approach of NOD/scid mouse tumor xenografts *in vivo* leading to an attenuated tumor growth. The differences in epothilone B concentrations used *in vitro* and *in vivo* can be related to protective effects by the tumor stroma *in vivo*. Thus, the tumor cells *in vitro* can be exposed directly to the drug whereas *in vivo*, the tumor microenvironment of extracellular matrix with embedded adjacent cell populations including immune cells, endothelial cells, cancer-associated fibroblasts and mesenchymal stem cells contribute to a border which requires higher chemotherapeutic concentrations to target the tumor cells [12,13].

In this context, it is remarkable to note that the closely related compound ixabepilone, which differs in only one atom by the exchange of oxygen against nitrogen within the ester bridge of the molecule, displayed no detectable effects on SCCOHT-1 cell growth and viability even at a 250-fold higher concentration as compared to epothilone B. These significantly different cellular effects of structurally very similar compounds are related to molecular differences in the interactions with tubulins and the associated stability of the microtubules. Whereas tubulinα and tubulinβ proteins associate to a heterodimer and form a taxane binding pocket at intradimer interfaces, epothilone B binding to this taxane site exhibits a tight interaction with the heterodimeric tubulin molecule and a rather static conformation. In contrast, ixabepilone retains a significant degree of flexibility within the atomic and molecular environment of this taxane binding pocket and therefore displays different effects on the interaction and stability with the tubulin heterodimeric molecule [29]. Such molecular interactions may also apply to the isotype form tubulinβ3 which is present predominantly in aggressive and drug-resistant tumors [30,31]. Indeed, a markedly detectable expression of tubulinβ3 has been identified in SCCOHT-1 cells in contrast to the NIH:OVCAR-3 and SK-OV-3 ovarian cancer cells which substantiates the aggressiveness of SCCOHT.

The significant epothilone B-mediated growth reduction of SCCOHT-1 cells *in vitro* was maintained in re-cultured cells from induced mouse xenograft tumors and these findings could also be confirmed *in vivo* demonstrating a tumor reduction by epothilone B in SCCOHT-1-induced mouse xenografts. Moreover, the tumor-reducing effects of epothilone B could be synergistically enhanced by exogenous calcium *in vitro* and *in vivo* and resulted in an attenuated tumor growth. The concomitant reduction of the hypercalcemic serum levels back to normal calcium serum levels observed in calcium + epothilone B-treated mice in contrast to the sustained hypercalcemia in untreated and solely calcium-treated tumors appear somewhat paradoxical and suggests an important but so far unexplained physiological role of calcium in this tumor entity. Since increased calcium levels can exhibit cytotoxic effects in ovarian cancer cells *in vitro* [32], hypercalcemia may partially represent a defense mechanism of the organism to antagonize the rapid and aggressive tumor growth and exogenously added calcium may further raise these levels for a sufficient synergy with epothilone B. However, the underlying mechanisms of this synergism remain unanswered and require further investigation.

## Conclusion

Whereas only little therapeutic strategies for unresponsive ovarian carcinoma are available, the present findings provide a more detailed understanding of potential compounds to target ovarian cancer cells exhibiting resistance to a variety of chemotherapeutics. Moreover, this work demonstrates a promising disease-focused approach including some molecular explanation for targeting the small cell carcinoma of the ovary hypercalcaemic type which may be embedded into a multi-modality therapeutic approach for a better targeted treatment of this rare cancerous disease.

## Abbreviations

SCCOHT: Small cell carcinoma of the ovary hypercalcemic type
GFP: Green fluorescent protein
PKD: Protein kinase D
